# Interventions targeting social determinants of mental disorders and the Sustainable Development Goals: A systematic review of reviews

**DOI:** 10.1192/j.eurpsy.2024.591

**Published:** 2024-08-27

**Authors:** T. K. Oswald, M. T. Nguyen, L. Mirza, C. Lund, H. G. Jones, G. Crowley, D. Aslanyan, K. Dean, P. Schofield, M. Hotopf, J. Das-Munshi

**Affiliations:** ^1^Department of Psychological Medicine, Institute of Psychiatry, Psychology & Neuroscience, King’s College London, London, United Kingdom; ^2^Discipline of Psychiatry and Mental Health, School of Clinical Medicine, Faculty of Medicine and Health, University of New South Wales, Sydney, Australia; ^3^University Hospitals Sussex, Sussex; ^4^Centre for Global Mental Health, Health Service and Population Research Department, Institute of Psychiatry, Psychology & Neuroscience, King’s College London, London, United Kingdom; ^5^Alan J Flisher Centre for Public Mental Health, Department of Psychiatry and Mental Health, University of Cape Town, Cape Town, South Africa; ^6^South London and Maudsley NHS Foundation Trust, London, United Kingdom; ^7^Justice Health and Forensic Mental Health Network, New South Wales, Australia; ^8^School of Life Course and Population Sciences, Faculty of Life Sciences and Medicine, King’s College London, London, United Kingdom

## Abstract

**Introduction:**

Globally, mental disorders account for almost 20% of disease burden and there is growing evidence that mental disorders are associated with various social determinants. Tackling the United Nations Sustainable Development Goals (UN SDGs), which address known social determinants of mental disorders, may be an effective way to reduce the global burden of mental disorders.

**Objectives:**

To examine the evidence base for interventions that seek to improve mental health through targeting the social determinants of mental disorders.

**Methods:**

We conducted a systematic review of reviews, using a five-domain conceptual framework which aligns with the UN SDGs (PROSPERO registration: CRD42022361534). PubMed, PsycInfo, and Scopus were searched from 01 January 2012 until 05 October 2022. Citation follow-up and expert consultation were used to identify additional studies. Systematic reviews including interventions seeking to change or improve a social determinant of mental disorders were eligible for inclusion. Study screening, selection, data extraction, and quality appraisal were conducted in accordance with PRISMA guidelines. The AMSTAR-2 was used to assess included reviews and results were narratively synthesised.

**Results:**

Over 20,000 records were screened, and 101 eligible reviews were included. Most reviews were of low, or critically low, quality. Reviews included interventions which targeted sociocultural (n = 31), economic (n = 24), environmental (n = 19), demographic (n = 15), and neighbourhood (n = 8) determinants of mental disorders. Interventions demonstrating the greatest promise for improved mental health from high and moderate quality reviews (n = 37) included: digital and brief advocacy interventions for female survivors of intimate partner violence; cash transfers for people in low-middle-income countries; improved work schedules, parenting programs, and job clubs in the work environment; psychosocial support programs for vulnerable individuals following environmental events; and social and emotional learning programs for school students. Few effective neighbourhood-level interventions were identified.

**Conclusions:**

This review presents interventions with the strongest evidence base for the prevention of mental disorders and highlights synergies where addressing the UN SDGs can be beneficial for mental health. A range of issues across the literature were identified, including barriers to conducting randomised controlled trials and lack of follow-up limiting the ability to measure long-term mental health outcomes. Interdisciplinary and novel approaches to intervention design, implementation, and evaluation are required to improve the social circumstances and mental health experienced by individuals, communities, and populations.

**Disclosure of Interest:**

None Declared

